# The GOLMePsA study protocol: an investigator-initiated, double-blind, parallel-group, randomised, controlled trial of GOLimumab and methotrexate versus methotrexate in early diagnosed psoriatic arthritis using clinical and whole body MRI outcomes

**DOI:** 10.1186/s12891-017-1659-1

**Published:** 2017-07-18

**Authors:** Gabriele De Marco, Philip Helliwell, Dennis McGonagle, Paul Emery, Laura C. Coates, Elizabeth M. A. Hensor, Helena Marzo-Ortega

**Affiliations:** 1 NIHR Leeds Biomedical Research Centre, Leeds Teaching Hospitals NHS Trust, Leeds, West Yorkshire UK; 2 Leeds Institute of Rheumatic and Musculoskeletal Medicine, University of Leeds, Chapel Allerton Hospital, 2nd Floor Chapeltown Road, Leeds, West Yorkshire LS7 4SA UK

**Keywords:** Psoriatic arthritis, Early diagnosis, Treatment-näive, TNF-inhibitor, Treat-to-target, Minimal disease activity, Time-to-recurrence

## Abstract

**Background:**

Psoriatic arthritis (PsA) is a chronic inflammatory arthritis which impacts significantly on the quality of life and work capacity of affected individuals. Recent evidence has shown that early control of inflammation in PsA leads to improved long-term outcomes. It is postulated that prompt intervention after diagnosis using a remission-induction treatment strategy will lead to improved outcomes and optimal disease control of PsA.

The aim of the present study was to compare the clinical efficacy of a treatment strategy in newly diagnosed, treatment naïve PsA subjects, using the combination of golimumab (GOL), methotrexate (MTX) and steroids versus standard care (MTX monotherapy plus steroids).

**Methods/design:**

GOLMePsA is an investigator initiated, phase IIIb, single-centre, randomised, double-blind, placebo-controlled, two-armed, parallel-group, imaging-supplemented study. Eighty-eight PsA patients, diagnosed within 24 months prior to screening and treatment naïve, will be randomised at baseline to receive: (arm 1) the combination of intramuscular/intra-articular prednisolone, MTX and GOL or (arm 2) the combination of intramuscular/intra-articular prednisolone, MTX and placebo for 24 weeks (interventional period). Primary outcome measure is clinical improvement (at least 1 unit difference) in the Psoriatic ArthritiS Disease Activity Score (PASDAS) composite index.

Reflecting a “step down” therapeutic approach, all participants successfully completing the interventional period will be followed up for a further 28 weeks. During this observational period, stable maintenance MTX monotherapy will continue for both arms, unless in case of intolerance or PsA relapse. In the latter case, additional treatment will be provided. Overall, the GOLMePsA study length is planned to be 52 weeks.

**Discussion:**

The hypothesis underlining this study is that very early treatment with first-line GOL reduces disease activity in PsA, in comparison to conventional therapy.

**Trial registration:**

EudraCT 2013–004122-28. 24/09/2013.

## Background

Psoriatic arthritis (PsA) is a polygenic, chronic inflammatory arthritis affecting up to 0.19% of the general population [1] and 3.2% [2] to 34% [3] of individuals with skin psoriasis. The course of PsA is heterogeneous and variable: some patients have mild disease whilst others evolve into a severe arthropathy that is often refractory to conventional treatments. These cases are frequently associated with functional disability and accelerated morbidity [4, 5]. A main hindrance in the treatment of PsA has been the delay to presentation to rheumatology clinics, of up to 9 years from symptoms onset. By that time the majority of affected individuals (67%) has an established, erosive arthropathy with a substantial degree of functional impairment [6–8]. However, up to 27% of PsA cases show radiographic damage within 2 years of symptom onset [9, 10]. Even a short delay of 6 months to presentation may lead to permanent work instability [11].

Over the past two decades, mounting evidence from rheumatoid arthritis (RA) studies has shown that early, aggressive treatment using synthetic and biologic disease-modifying anti-rheumatic drugs (sDMARDs and bDMARDs, respectively) improves the outcome of the disease concerning articular damage and disability [12]. Such a novel therapeutic approach allowed clinical remission to become an achievable goal in many patients treated with bDMARDs, chiefly Tumor Necrosis Factor α (TNFα) inhibitors (TNFi) [13, 14]. As a result, the concepts of “window of opportunity” and “treat-to-target” have become popular in RA treatment. By contrast, data in PsA are still sparse [15], with only limited evidence on the efficacy of sDMARDs such as methotrexate (MTX) [16] or leflunomide [17]. To date, only TNFi have shown efficacy on all clinical manifestations of PsA [18–21] as well as on delaying structural damage [22]. The safety and efficacy of golimumab (GOL) used for reducing signs and symptoms of active PsA and the associated skin and nail disease was evaluated in the GO-REVEAL trial) [19] with benefits maintained in the long term [23]. Further, in the same trial GOL was associated with inhibition of structural damage (weeks 24 and 52), persistent articular and cutaneous improvements at week 104 and significant improvements in the Patients’ Reported Outcomes (PROs). Currently, GOL, alone or associated with MTX, is indicated to treat active and progressive PsA in adults irresponsive or intolerant to previous sDMARD therapy.

Structural abnormalities are insidious in the early disease stages of PsA, rendering radiography a measurable outcome lacking of sensitivity in these patients. Whole body MRI (WB-MRI) allows axial and peripheral multi-joint assessments in one single investigation and is a feasible tool for detection of subclinical inflammation in patients with PsA, particularly for enthesitis and bone marrow oedema (BMO) [24]. Evidence from RA suggests that subclinical BMO is one of the main predictors for on-going radiographic progression, even in the presence of clinical remission [25]. In SpA, there is evidence that BMO may be a predictor of future radiographic axial damage [26]. The ability of WB-MRI and US [27, 28] to identify subclinical inflammation is particularly useful for the quantification of the total inflammatory burden in PsA [29] and in understanding the features of disease remission after a course of therapy.

To date, there are no data on the role of WB-MRI as an outcome measure in PsA. Further, the use of bDMARDs on treatment naïve, early diagnosed PsA patients has not been addressed in double blind randomized clinical trials. It is therefore postulated that the GOLMePsA study will show that an early aggressive intervention in PsA immediately after diagnosis, using GOL combined with dose escalating MTX protocol and intra-articular or intramuscular corticosteroid, could ameliorate significantly the disease activity and even lead to a state of minimal disease activity (MDA) [30], or near clinical remission, along with complete ablation of inflammation as shown by WB-MRI at 24 weeks.

### Study aims

The aim of this study is to assess in early diagnosed treatment naïve PsA, the clinical efficacy of a treatment strategy comprising of the combination of GOL plus MTX plus steroids versus standard care (MTX monotherapy plus steroids) using clinical and imaging outcome measures at 24 weeks.

## Methods

### Research hypothesis

The GOLMePsA trial was designed to address two different hypotheses:

- First, that early intervention in recently diagnosed and treatment-näive PsA, through the combination of GOL and MTX plus corticosteroids will lead to clinical improvement reflected by at least 1 unit difference on the scale measured by the Psoriatic ArthritiS Disease Activity Score (PASDAS) [31–34] at week 24. The improvement will be superior to that shown by the combination of MTX plus placebo plus corticosteroids (conventional therapy).
- Secondly, that a subset of PsA patients at presentation have a substantial amount of subclinical articular and/or entheseal inflammation and this can be detected by WB-MRI and US. Using these imaging techniques will allow the identification of subjects whose response to therapy has been successful even at the subclinical level.

### Primary objective

The primary objective of this study is to assess whether the combination of GOL with MTX and steroids is superior to standard care (MTX monotherapy plus steroids) in patients with early treatment naïve PsA using the PASDAS score at 24 weeks as the primary outcome measure.

### Secondary objective(s)

- To assess the extent of association between clinical and imaging joint assessments at baseline.
- To assess the extent of association between clinical and imaging responses to therapy.
- To assess whether responses on imaging outcomes are associated with steroid therapy.
- To assess the superiority of combination therapy over standard treatment in improving patient-reported QoL and health status.
- To identify baseline variables which may be modifiers of clinical or imaging response (e.g.: symptom duration, immunological parameters).

### Trial design

GOLMePsA is an investigator initiated, phase IIIb, single-centre, randomised, double-blind, placebo-controlled, two-armed, parallel-group, imaging-supplemented study.

A total of 88 patients with PsA, diagnosed within 24 months prior to screening and treatment näive, will be randomised to compare:

- The combination of intramuscular/intra-articular prednisolone and MTX plus GOL

TO

- The combination of intramuscular/intra-articular prednisolone and MTX plus placebo

**Fig. 1:**
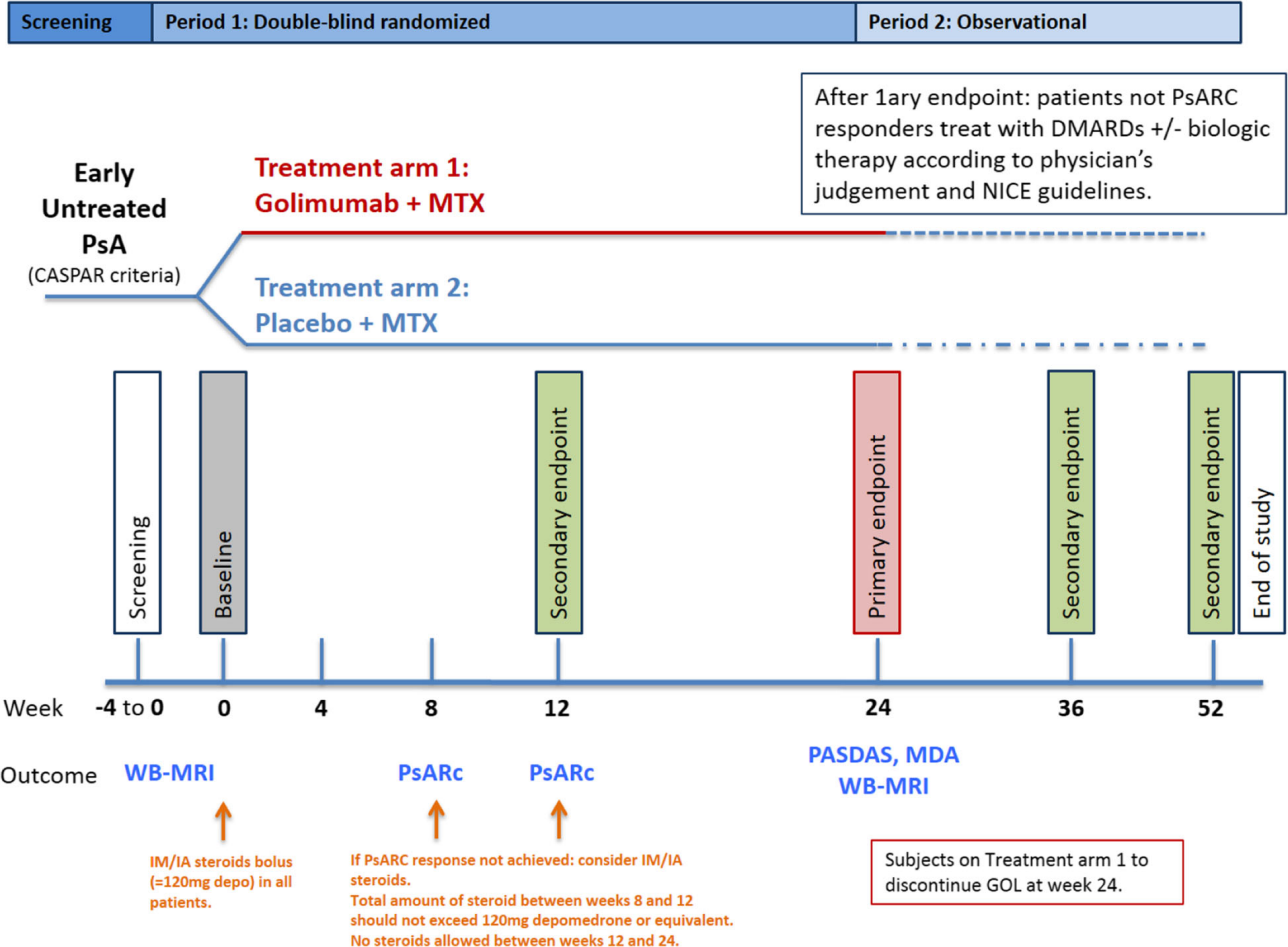
Schematic diagram of screening, randomisation and treatment procedures of the GOLMePsA trial. PsARC = Psoriatic arthritis response criteria; DMARD = Disease modifying anti-rheumatic drug; NICE = National Institute for Health and Care Excellence; PsA = Psoriatic arthritis; MTX = Methotrexate; CASPAR = ClASsification criteria for Psoriatic Arthritis [39]; WB-MRI = Whole-body magnetic resonance imaging; PASDAS = Psoriatic arthritis disease activity score; GOL = Golimumab

**Fig. 2:**
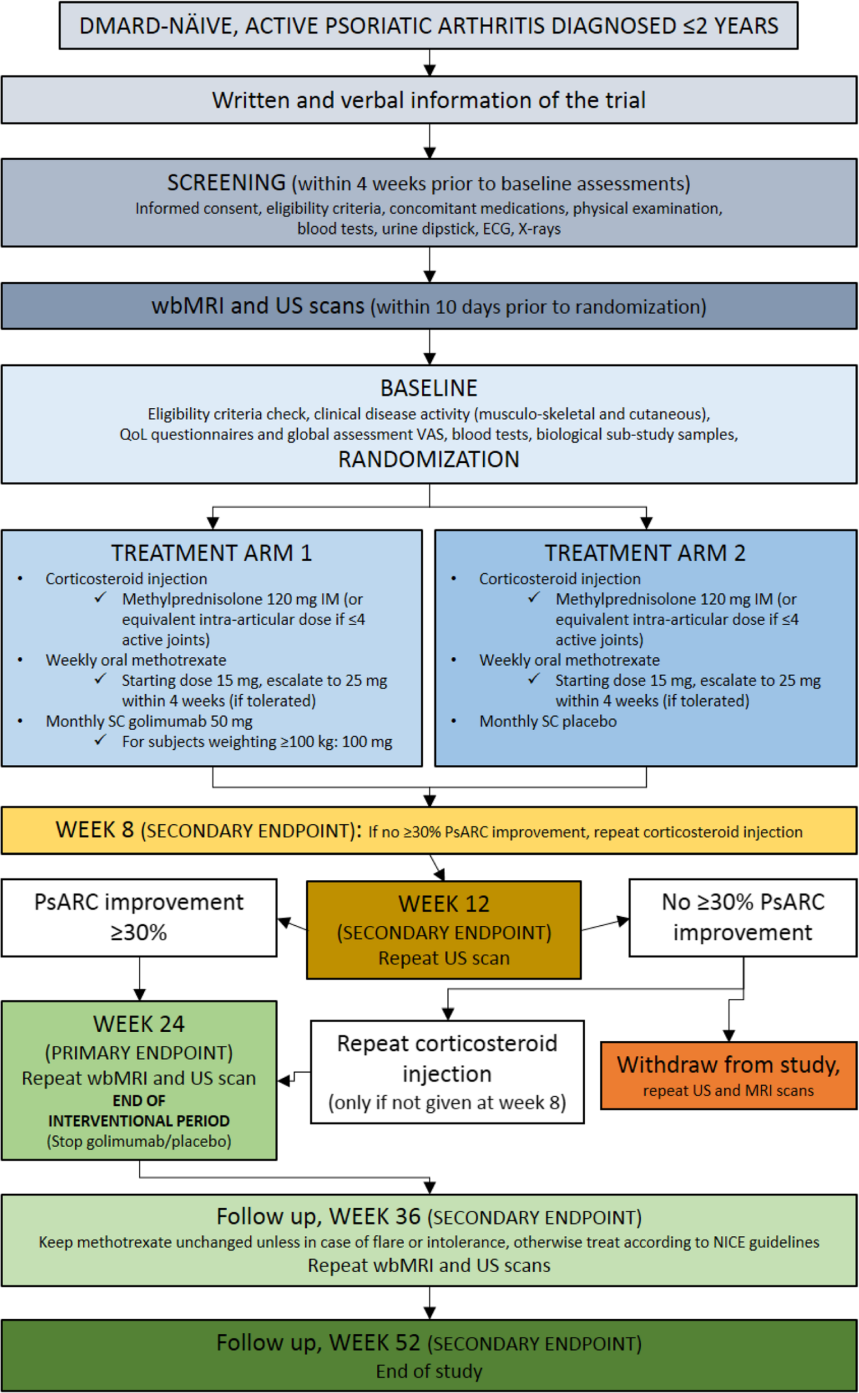
GOLMePsA trial flow diagram. DMARD = Disease-Modifying Anti-Rheumatic Drug; ECG = Electrocardiogram; wbMRI = Whole body magnetic resonance imaging; US = Ultrasound; QoL = Quality of life; VAS = Visual Analogue Scale; IM = Intra-muscular; SC = Subcutaneous; PsARC = Psoriatic arthritis response criteria

The above described interventional period will last 24 weeks. All participants successfully completing this phase will be followed up for other 28 weeks (observational period). Overall, the GOLMePsA study length is planned to be 52 weeks (see Figs. 1 and 2).

### Eligibility

The target population is early diagnosed (within 2 years from screening date), treatment naïve PsA cases who satisfy the ClASsification criteria for Psoriatic ARthritis (CASPAR) [35]. A full list of inclusion and exclusion criteria for eligibility and subsequent randomization into this study are detailed in Table 1.

**Table 1:** Eligibility criteria for randomization into the GOLMePsA trial

Inclusion criteria	
1	Male and female subjects aged ≥18 years at the time of signing the Informed Consent Form.
2	Patients with a diagnosis of PsA and fulfilling CASPAR classification criteria confirmed within 24 months prior to the screening visit.
3	Patients with active PsA, defined by: • the presence of at least 3/68 tender joints AND at least 3/66 swollen joints; OR • 2 swollen AND 2 tender joints, along with one affected entheseal site (Achilles tendon and/or plantar fascia).
4	Subjects capable of understanding and signing an Informed Consent Form prior to any trial-related procedure.
5	Women of childbearing potential (WCBP) or men capable of fathering children must be using adequate birth control measures (e. g.: abstinence, oral contraceptives, intrauterine device, barrier method with spermicide, surgical sterilization) during the study and for 6 months after receiving the last administration of study drugs. WCBP have to test negative for pregnancy. Female subjects must agree to not donate eggs (ova, oocytes) during the study and for 6 months after last dose of study agent. Male subjects must agree to not donate sperm while in the study and for 6 months after last dose of study agent.
6	Patients with active current or latent tuberculosis (TB), including those diagnosed as a result of GOLMePsA trial screening procedures, who can provide adequate documentation of previous or were recently commenced on adequate anti-TB treatment according to local practice guidelines prior to the start of protocol treatment.
Exclusion criteria	
*General*	
7	Planned surgery within the study period which is expected to require omission of any study medication of 28 days or more
*Study specific*	
8	Patient who have received previous treatment with any sDMARD.
9	Patient who have received previous treatment with golimumab or other TNFi or other biologic or investigational drugs.
10	Any chronic inflammatory arthritis diagnosed before 16 years of age.
11	Patients with current crystal or septic arthritis.
12	The candidates ineligible to (see Table 4) or unsuccessful in bearing the WB-MRI procedures will not be excluded from the study.
*Excluded or concomitant therapy*	
13	Patient who have received any corticosteroids within 4 weeks prior to screening.
*Exclusions for general safety*	
14	Patients with significant concurrent medical conditions including: • Uncompensated congestive heart failure; • Myocardial infarction within 52 weeks from screening; • Unstable angina pectoris; • Uncontrolled arterial hypertension (blood pressure > 160/95 mmHg); • Severe pulmonary disease; • History of human immunodeficiency virus infection or immunodeficiency syndromes; • Central nervous system demyelinating events suggestive of multiple sclerosis; • Renal or gastrointestinal conditions; Which in the opinion of the investigator place the patient at an unacceptable risk for participation in the study or would make implementation of the protocol difficult.
15	Patients with cancer or a history of cancer (other than resected cutaneous basal cell carcinoma and in situ uterine cervical cancer) within 5 years of screening.
16	Patients with chronic infections of the upper respiratory tract (e. g.: Sinusitis), chest (e. g.: Bronchiectatic lung disease), urinary tract or skin (e. g.: Paronychia, chronic ulcers, open wounds) within 4 weeks of screening.
17	Patients who have a chest radiograph within 3 months prior to the first administration of study agent that shows an abnormality suggestive of a malignancy or current active infection, including TB (for TB exceptions refer also to inclusion criteria 6), histoplasmosis or coccidioidomycosis.
18	Patients with any ongoing or active infection or any major episode of infection requiring hospitalization or treatment with IV antibiotics within the preceding 30 days of screening and/or orally administered antibiotics in the preceding 15 days of screening.
19	Patients with abnormal liver function including known liver cirrhosis, fibrosis, or known non-alcoholic steato-hepatitis at the time of screening or abnormal blood tests as shown by: • Aspartate aminotransferase / alanine aminotransferase >3× upper limit of normality, OR • Bilirubin >51 μmol/L.
20	Patients with known severe hypoproteinaemia at the time of screening, e. g. in nephrotic syndrome or impaired renal function, as shown by: • Serum Creatinine >133 μmol/L.
21	Patients with known significantly impaired bone marrow function, e. g. significant anaemia, leukopaenia, neutropaenia or thrombocytopaenia, as shown by the following laboratory values at the time of screening: • White blood cells <3000 × 10^6/L; • Platelets <125 × 10^9/L; • Haemoglobin <90 g/L for males and <85 g/L for females.
22	Patients with a history of untreated latent or active TB prior to screening will not be eligible (for exceptions refer to inclusion criteria 6).
23	Subjects must undergo screening for hepatitis B virus (HBV). At a minimum, this includes testing for HBsAg (surface antigen), anti-HBs (surface antibody), and anti-HBc total (core antibody total). • Subjects who test positive for surface antigen (HBsAg+) are not eligible for this study, regardless of the results of other hepatitis B tests. • Subjects who test negative for surface antigen (HBsAg-) and test positive for core antibody (anti-HBc+) and surface antibody (anti-HBs+) are eligible for this study. • Subjects who test positive only for surface antibody (anti-HBs+) are eligible for this study. • Subjects who test positive only for core antibody (anti-HBc+) must undergo further testing for HBV deoxyribonucleic acid (HBV DNA test). If the HBV DNA test is positive, the subject is not eligible for this study. If the HBV DNA test is negative, the subject is eligible for this study. In the event the DNA test cannot be performed, the subject is not eligible for the study.
24	Primary or secondary immunodeficiency (history of or currently active) unless related to primary disease under investigation.
25	Pregnancy, lactation (nursing) or WCBP unwilling to use an effective birth control measure (detailed in the inclusion criteria 5) whilst receiving treatment and after the last dose of protocol treatment as indicated in the relevant Summary of Product Characteristics (SmPC)/Investigator Brochure (IB).
26	Men unwilling, or whose partners are WCBP who are unwilling to use an effective birth control measure (detailed in the inclusion criteria 5) whilst receiving treatment and after the last dose of protocol treatment as indicated in the relevant SmPC/IB.
27	Patients with a history of confirmed blood dyscrasia.
28	Patients with a history of mental illness that would interfere with their ability to comply with the study protocol.
29	Patients with a history of drug and/or alcohol abuse that would interfere with their ability to comply with the study protocol.
30	Patients with a history of any viral hepatitis within 1 year of screening.
31	Patients who have received or are expected to receive any live virus or bacterial vaccinations or treatments that include live organisms (e. g.: a therapeutic infectious agent such as the bacillus of Calmette-Guerin (BCG) that is instilled into the bladder for the treatment of cancer) within 3 months prior to the first administration of the investigational medicinal product (IMP) and/or non investigational medicinal products (NIMPs), during the trial, or within 6 months after the last administration of the IMP and/or NIMPs.
32	Patients who demonstrate hypersensitivity to the IMP and/or NIMPs, or any of the excipients detailed in the relevant SmPC.

*sDMARD* synthetic disease modifying anti-rheumatic drug, *TNFi* Tumor necrosis factor α inhibitor, *WB-MRI* whole-body magnetic resonance imaging

### Recruitment

This study is being conducted at the Chapel Allerton Hospital (CAH) Outpatient Department and Research Facility, part of the Leeds Teaching Hospitals National Health Service (NHS) Trust in Leeds, United Kingdom. Potential study candidates may also be identified via rheumatology clinics at Participant Identification Centres (PICs) within the Yorkshire Region (see also acknowledgements section). Potential candidates are provided with verbal and written details about the trial (Participant Information Sheet and Informed Consent Document) before being contacted by the main research team based at CAH. Potential candidates have as long as they need to consider participation. Assenting subjects are invited to provide informed, written consent before being registered into the trial and formally assessed for eligibility.

### Consent to the GOLMePsA trial biological sub-study

Eligible subjects are also invited to take part in a Biological Sub-study which collects biological samples (blood and urine) at predefined endpoints. Assenting subjects are asked to sign an additional, specific consent form.

### Screening and registration

**Fig. 3:**
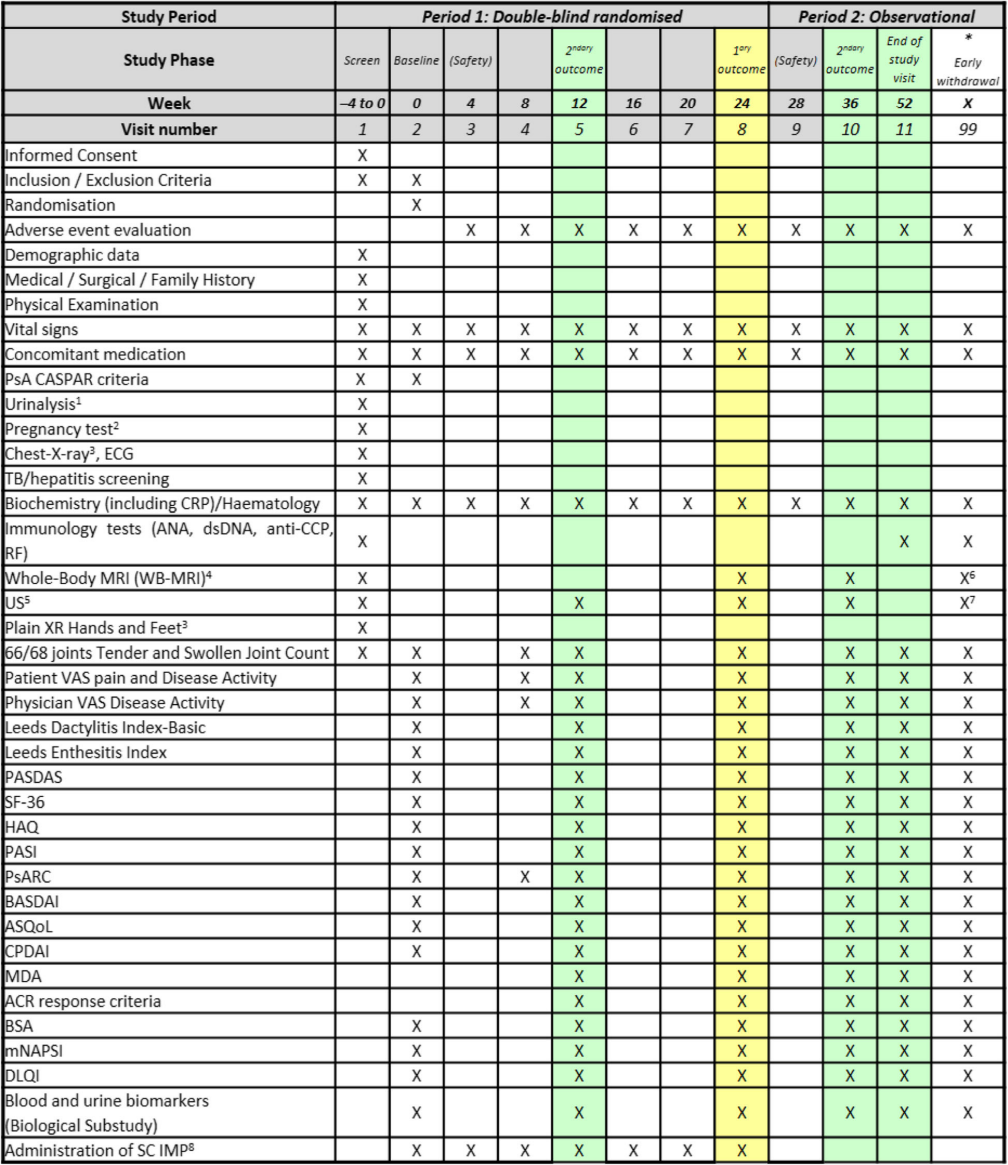
Summary schedule of study assessments. 1, 2: Urinalysis and Pregnancy test can be repeated in other visits as clinically indicated. 3: If subjects do not have a chest x-ray or hands/ft x-ray performed within 3 months of screening, an x-ray should be performed after it is certain the subject meets the inclusion/exclusion criteria in order to minimize exposure to ionising radiation. 4, 5: Whole-body magnetic resonance imaging (WB-MRI) and ultrasound (US) scans should be performed within 10 days before or after the scheduled visit attendance. Baseline assessment can take place 10 days before, but not after, the scheduled visit attendance. 6, 7: No imaging (WB-MRI and/or US) to be performed if withdrawal visit occurs after week 36 or if within 6 weeks of last imaging. 8. Investigational Medicinal Product (IMP) administration should be every 4 weeks. In the case of a missed dose of IMP, the IMP can be administered up to 2 weeks after the scheduled visit. If a dose of IMP is delayed for more than 2 weeks, the IMP should not be administered until the next scheduled visit. Exposure to IMP should be captured in the medication workbook. * Study week X: withdrawal or early discontinuation. Subjects who discontinue prematurely during Period 1 should return for the same assessments associated with Week 24 visit

Following written informed consent and prior to any trial-related procedures, participants are registered in the study enrolment log. All subjects undergo a screening assessment (Figs. 2 and 3) to determine eligibility for the study within 4 weeks prior to the baseline assessments.

The research team keeps a “pre-screening” log with details of all subjects who have been considered for the trial, regardless of final ineligibility or declined participation. These subjects are all referred back to outpatient rheumatology clinics in order to receive standard NHS care.

### Randomisation

Following registration, confirmation of eligibility, imaging investigations and completion of baseline assessments and questionnaires, participants are randomised in a 1:1 ratio into one of the two treatment arms. Randomization assignment is performed with a computer-based program which utilizes randomly-permuted block sizes. Randomisation is stratified by the number of clinically involved joints (oligoarticular: ≤4 joints/polyarticular: >4 joints).

### Trial interventions

Treatment is administered in the two study arms as detailed in Table 2. The planned use of corticosteroids, given as a “bolus” at baseline to all participating subjects, aims to achieve rapid ablation of inflammation. Participants in both arms not achieving at least 30% improvement in the Psoriatic Arthritis Response Criteria (PsARC) [36, 37] at weeks 8 and 12 will receive additional corticosteroid injections. The steroid amount given between both visits will not exceed a maximum of 120 mg methylprednisolone per patient (or comparable amount intra-articularly; unless contraindicated or not tolerated). No further steroids will be allowed between week 12 and week 24. All participants, in both arms, not achieving at least 30% of PsARC improvement beyond week 12 and before week 24 may discontinue the study medication regimen and be treated as clinically indicated. In this case, they may be withdrawn from the treatment part of the study.

**Table 2:** Description of the two treatment arms planned for the GOLMePsA trial

Treatment arm		Treatment description
1	Golimumab (IMP)	Monthly subcutaneous dose of 50 mg to be administered at the study site on baseline, week 4, 8, 12, 16, 20 and 24. Subjects of ≥100 kg in weight will be given golimumab 100 mg monthly.
	Methotrexate (NIMP)	Starting oral dose of 15 mg weekly at baseline. If tolerated, all participants will increase the weekly dose to 20 mg and 25 mg at weeks 2 and 4, respectively. The drug will be kept at 25 mg, or the highest tolerated oral weekly dose, until the end of the study. Subjects intolerant to oral formulation will switch to the subcutaneous one.
	Methylprednisolone (NIMP)	Single intra-muscular injection of 120 mg at baseline (or equivalent amount intra-articularly in case of oligoarticular presentation, defined by the presence of ≤4 swollen joints).
	Folic acid (NIMP)	Daily oral dose of 5 mg, 6 days per week (except the day of methotrexate), until the end of the study.
2	Placebo	Monthly subcutaneous administration at the study site on baseline, week 4, 8, 12, 16, 20 and 24.
	Methotrexate	As described for treatment arm 1
	Methylprednisolone	As described for treatment arm 1
	Folic acid	As described for treatment arm 1

*Abbreviations*: *IMP* Investigational medicinal product, *NIMP* Non-investigational medicinal product

All subjects completing the interventional period will continue their MTX and folic acid supplementation unchanged through the observational period if tolerated. During the observational period, in case of increase in disease activity (PsA flare) compared to the previous assessments, the treating physicians will be allowed to consider other available NHS therapeutic options for PsA, such as corticosteroids, sDMARDs or bDMARDs.

After the end of the interventional period and before the end of the trial, intramuscular or intra-articular steroids are not permitted if a clinical or imaging assessment is scheduled within the following 6 weeks.

### Assessments, samples and data collection

All protocol-related assessments are recorded on paper source data sheets (SDS) and stored at the research site. These data are then entered onto an electronic case report form (eCRF) specific for the GOLMePsA trial. The trial visits are structured as detailed in Fig. 3.

Biological samples from participants consenting to the GOLMePsA trial Biological Substudy are collected at baseline prior to commencement of trial treatment followed by weeks 12, 24, 36 and 52. In addition, biological samples are collected at the time of early discontinuation (withdrawal visit) if this falls outside the pre-specified time-points (see Fig. 3). Personal details are removed from all biological samples collected as part of the Biological Substudy, after separation into their component parts and before testing. For storage and testing purposes, a pseudo-anonymization unique code will be generated to link the clinical, imaging and laboratory databases. All the biological samples are stored in the central GOLMePsA Trial Biobank in a laboratory at our institution. These samples will be used for a range of studies of direct relevance to the treatment of PsA.

### Outcome measures

#### Clinical efficacy

The primary outcome in this study is the change in PASDAS score at 24 weeks. The PASDAS is a weighted composite index encompassing joints counts, an assessment of enthesitis and dactylitis, acute phase reactant, QoL, and patients’ and physician’s global assessment by visual analogue scale (VAS). The PASDAS has been shown to perform well in both oligoarticular and polyarticular forms of PsA [36] and cut-offs for disease activity and response have now been developed and validated using interventional trial data [34].

Secondary outcomes at weeks 12, 24, 36 and 52 include the Leeds enthesitis index (LEI) [38], the Leeds dactylitis index basic (LDI-B) [39, 40], the Psoriasis Area and Severity Index (PASI) score [41], the Body Surface Area (BSA) affected by psoriasis, the modified Nail Psoriasis Severity Index (mNAPSI) [42], the proportion of subjects achieving MDA, the proportion of subjects achieving the American College of Rheumatology Response Criteria [43], the proportion of subjects achieving the PASI75 response (defined as having an improvement of at least 75% in the PASI score compared to baseline levels). The proportion of subjects achieving PsARC response is a secondary outcome to be collected at weeks 8, 12, 24, 36 and 52. The Composite Psoriatic Disease Activity Index (CPDAI) [44] is a secondary outcome to be collected at weeks 24 and 52. The proportion of patients requiring additional steroid therapy, as well as the cumulative steroid dose up to week 12 of this trial, will be also recorded as a secondary outcome.

#### Imaging measures of disease activity

Systematic WB-MRI scanning of the axial and peripheral skeleton (Table 3) is performed in all suitable subjects (Table 4), using commercially available Siemens MAGNETOM® Verio 3 T scanner with the following sequences: (i) T1-weighted spin echo (SE) before and after an intravenous gadolinium contrast injection; (ii) Short Tau Inversion Recovery (STIR). The estimated scanning time is 60 min. Images are acquired in two phases, allowing patients to mobilize from the scanner for a short time. MRI features of inflammation (ie: synovitis, BMO or osteitis lesions) and damage (erosions, bone formation, fat infiltration, sclerosis and/or ankylosis) will be scored at peripheral joints, entheses and axial skeleton including sacroiliac joints and spine using a novel scoring system.

**Table 3:** Body areas undergoing magnetic resonance imaging (MRI) and ultrasound (US) evaluation

*Articular Site*	*Number of joints*	*MRI*	*US*
Spine	42	X	
Sacro-Iliac joints (SIJ)	2	X	
Acromion-clavicular joints	2	X	
Wrists	2	X	X
Metacarpo-phalangeal joints (from 1 to 5)	10	X	X
Proximal interphalangeal joints of the hands (from 1 to 5)	10	X	X
Distal interphalangeal joints of the hands (2–5)	8	X	
Hips	2	X	
Knees	2	X	X
Ankles	2	X	X
Mid/Hind foot	2	X	
Metatarso-phalangeal joints (from 1 to 5)	10	X	X
*Entheseal Site*	*Number of areas*		
Lateral humeral epicondyle	2		X
Quadriceps insertion onto patella	2		X
Medial Femoral Condyle	2	X	
Proximal patellar ligament insertion	2		X
Distal patellar ligament insertion	2		X
Achilles’ tendon distal insertion	2	X	X
Plantar fascia proximal insertion	2	X	X

**Table 4:** Eligibility criteria to gadolinium contrast-enhanced magnetic resonance imaging at the Leeds Musculoskeletal Biomedical Research Unit

Absence of previous reactions to gadolinium contrast	
Absence of concomitant allergies to multiple drugs	
Absence of severe allergies to drugs or food	
Absence of a pacemaker	
Absence of metallic implants (e. g.: cardiac valves, joint prostheses, stents, cochlear implants)	
Absence of metallic fragments in the eyes	
Absence of unstable bronchial asthma	

All criteria must be satisfied to fulfil eligibility

US scanning of selected joints and entheses of lower and upper limbs (see Table 3), is performed using a multi-planar technique with symmetrical scanning by sonographer blinded to the participant’s clinical characteristics. Articular and entheseal sites will be assessed for the presence of grey scale (GS) abnormalities and power-Doppler (PD) signal. US pathological findings will be identified according to the Outcome Measures in Rheumatoid Arthritis Clinical Trials (OMERACT) definitions [45–47].

#### Patient-reported outcomes

The patients’ overall assessment of PsA activity will be recorded at baseline and then at weeks 12, 24, 36 and 52, using the 100 mm horizontal VAS using the specific wording proposed by Cauli et al. [48] for PsA. To calculate the PsARC (at weeks 8, 12, 24, 36 and 52), the same assessment will be also recorded on a Likaert scale ranging from 1 to 5. All participants will fill in the Bath Ankylosing Spondylitis Disease Activity Index (BASDAI) [49] questionnaire at baseline and then at weeks 12, 24, 36 and 52. The outcomes relating to QoL and health status are: the Health Assessment Questionnaire Disability Index (HAQ-DI) [50] score; the Dermatology Life Quality Index (DLQI) [51] score; the Ankylosing Spondylitis Quality Of Life (ASQOL) [52] score; the Short Form (SF-36) [53] score. All these questionnaires will be collected at baseline and then at 12, 24, 36 and 52 weeks.

#### Statistical analysis

Our null hypothesis (H0) is that the difference between the two treatment arms in the PASDAS score at week 24 is equal to zero. Hence, the alternative hypothesis (H1) is that the difference between the two treatment arms in the PASDAS score at week 24 is not equal to zero. In general, summary statistics (n = number of available measurements; arithmetic mean; standard deviation; median; minimum and maximum) for quantitative variables and absolute and relative frequency tables for qualitative data will be presented. Analyses will be adjusted for the randomisation stratification factor(s) and baseline values of the outcome; 2-tailed tests will be performed and will be considered statistically significant if *p* < 0.05.

#### Planned efficacy analyses

The primary endpoint will be assessed on an intention-to-treat (ITT) basis. Analysis of covariance by multiple linear regression will be used to compare PASDAS between the two treatment groups at week 24. Binary secondary endpoints will be analysed using multiple binary logistic regression. Continuous interval outcomes will be analysed using multiple linear regression. Severely skewed or ordinal outcomes will be analysed using quantile regression. Planned subgroup analyses will investigate differences in treatment response according to oligo/polyarthritis status, immunological status, disease duration at baseline. A per protocol analysis will also be performed.

#### Safety analyses

The frequency of all Serious Adverse Events (SAEs) during the study period in patients who received at least 1 dose of study treatment will be presented for each treatment group separately. The data will be displayed as number of subjects experiencing the SAEs, percentage of subjects, and number of SAEs. Data will also be corrected for exposure by 100 patient-years.

#### Handling of dropouts and missing data

For patients who withdraw early, data from the withdrawal visit will be imputed for subsequent visits for continuous outcomes, and non-response will be imputed for binary outcomes. For all other instances of missing data, multiple imputation will be used. A number of sensitivity analyses testing robustness of conclusions under different missing data mechanisms will be conducted.

#### Determination of sample size

Using in-house unpublished data, we estimated the minimum clinically important difference to be 0.7 units on the PASDAS; this is similar to a published value for smallest detectable difference of 0.8 units [34]. We will aim to detect a difference of at least 1 unit between the treatment arms in this study. The standard deviation of PASDAS in the Tight Control of Psoriatic Arthritis (TICOPA) [54] MTX rapid escalation arm at 24 weeks (restricted to patients who remained on methotrexate throughout) was 1.57. Assuming δ = 1, σ = 1.57, at alpha = 0.05 and 1-Beta = 0.8 this would require 78 patients; accounting for 10% drop-out we will aim to recruit a total of 88 (44 per group).

## Discussion

PsA is a costly disease both for the individual and the society. Current treatment guidelines and treat-to-target strategies are based on a step-up approach with sDMARDs being the main staple of treatment, despite limited data supporting their efficacy. The GOLMePsA study aims to test the hypothesis that rapid control of disease activity by aggressive abrogation of inflammation with bDMARDs (GOL) in early diagnosed PsA will lead to better outcomes at 24 weeks when compared to sDMARD. Observing both arms up to 52 weeks will allow us to investigate whether the anticipated benefit of early use of bDMARDs leads to sustained response while on MTX monotherapy and better disease control at the end of the observational period. In addition, rapid optimization of disease management is expected to lead to significant improvements in QoL of affected individuals. A recent post-hoc analysis of the PRESTA trial provided some evidence on the superior efficacy of early bDMARD use in PsA by showing that subjects treated within 2 years of clinical onset had greater improvements in disease activity outcomes and PROs [55] than those with a longer disease duration. Data from an observational Swedish cohort [15] have highlighted the link between shorter symptom duration at presentation and a favourable outcome at 5 year follow-up. The GOLMePsA study was conceived in parallel to the running of the TICOPA trial in our institution and before any results from the latter were available. Recent evidence published since from the TICOPA trial [54] supports the notion that early and intensive treatment ameliorates disease activity effectively. These findings support our initial hypothesis that shortening the time from symptom onset to diagnosis, coupled with an aggressive therapeutic approach, should provide a window of opportunity for optimal management of PsA. The GOLMePsA trial will compare two different treatment combination strategies for PsA (single corticosteroid injection plus MTX plus bDMARD versus single corticosteroid injection plus MTX). This translates into both treatment arms receiving active medication (MTX and corticosteroids). These interventions will allows us to gather more data concerning the efficacy of early intensive treatment strategies in PsA and to characterize the role of bDMARD therapy early in the course of the disease. This study design will also provide important insights concerning the efficacy and safety of a “step down” strategy with bDMARDs followed by MTX maintenance in contrast to a more cautionary “step up” approach (bDMARDs following sDMARDs failure or intolerance). The effect of adding sDMARDs to MTX in early PsA patients stepping down from bDMARDs will also be explored.

Further, the GOLMePsA trial is collecting data from several clinical manifestations of PsA (articular and cutaneous involvement, entheses, dactylitis and nail disease). The choice of PASDAS as primary outcome will contribute to explore issues of multidimensionality in a heterogeneous condition like PsA. From the regulatory/ethic point of view, however, PsARC was chosen to manage efficacy decisions at weeks 8–12 as a reflection of current treatment guidelines in the UK.

Further, more evidence will become available on the efficacy of high-dose MTX as initial agent in the treatment of the early phases of PsA. Previous studies exploring this issue have reported promising results [16, 56], although they could have potentially underestimated the presence of a dose effect. One of the main findings from the TICOPA trial was the apparently higher number of side effects reported on the tight control arm. The main difference between the GOLMePsA study and the TICOPA trial is the earlier exposure to bDMARDs as part of an aggressive combination strategy also incorporating methotrexate and steroids. This could lead to higher toxicity than the conventional treatment arm, although our experience, with nearly 25% of the GOLMePsA subjects recruited, has not raised such concerns so far.

Finally, GOLMePsA uses an imaging package comprising WB-MRI and US to assess the overall burden of inflammation in early PsA. This will allow for the characterization of bulk of subclinical disease as a possible biomarker of treatment response and prognosis at 24 and 36 weeks.

### Trial status

The first patient was enrolled in GOLMePsA in November 2015 with recruitment planned to end in April 2018. There are an estimated 4–6 eligible subject per month seen in our Early Arthritis and Spondyloarthritis clinics, yielding a possible 48 eligible patients per year. Since opening, the trial has undergone minor protocol amendments (current version 4.0). All these are reflected in the present paper.
